# The association between socioeconomic status and health-related quality of life among young and middle-aged maintenance hemodialysis patients: multiple mediation modeling

**DOI:** 10.3389/fpsyt.2023.1234553

**Published:** 2023-09-19

**Authors:** Qingxin Mai, Siyi Xu, Jingyi Hu, Xiaoming Sun, Gangyi Chen, Zhen Ma, Yang Song, Chao Wang

**Affiliations:** ^1^School of Nursing, Guangzhou University of Chinese Medicine, Guangzhou, China; ^2^Department of Nephrology, The First Affiliated Hospital of Guangzhou University of Chinese Medicine, Guangzhou, China; ^3^Department of Nephrology, Guangzhou Hospital of Integrated Traditional Chinese and Western Medicine, Guangzhou, China

**Keywords:** maintenance hemodialysis, socioeconomic status, health-related quality of life, illness perception, social functioning

## Abstract

**Objective:**

To explore the relationship between socioeconomic status (SES), illness perception, social functioning, and health-related quality of life (HRQoL) of young and middle-aged maintenance hemodialysis (MHD) patients and the internal mechanism of action.

**Design:**

A multicenter cross-sectional study.

**Methods:**

An aggregate of 332 young and middle-aged MHD patients were enrolled from hemodialysis centers in four general hospitals in Guangzhou, Guangdong, China, from June to December 2022. The questionnaires used included one for general demographic data, the Brief Illness Perception Questionnaire (BIPQ), Social Dysfunction Screening Scale (SDSS), and the 12-item Short Form Health Survey (SF-12).

**Results:**

Both SES and HRQoL were negatively correlated with illness perception and social functioning, respectively. SES was positively correlated with HRQoL. Illness perception was positively correlated with social functioning. The indirect effects of illness perception and social functioning on the relationship between SES and HRQoL were 0.33 and 0.31, making up 41.06% and 38.91% of the sum. The chain indirect effect of illness perception and social functioning was 0.10, making up 12.59% of the total effect, while gender did not play a moderating role.

**Conclusion:**

Illness perception and social functioning may independently and accumulatively mediate the association between SES and HRQoL. Nurses should consider developing individual intervention program for young and middle-aged MHD patients with low SES, focusing on establishing targeted counseling and health education strategies corresponding to illness perception and social functioning to help patients improve their HRQoL.

## 1. Introduction

The end-stage of various chronic kidney diseases (CKD) is also known as End-stage renal disease (ESRD). Approximately 4 million ESRD patients’ survival are dependent on renal replacement therapy, and it is expected to reach 5.4 million by 2030 (1). Maintenance hemodialysis (MHD) is the most prevalent renal replacement therapy, taking up about 69% of all renal replacement therapies and 89% of dialysis treatment since number and access to kidney donors are limited and peritoneal dialysis technology has its own limitations (2). In China, the rapid development of the socio-economic level is accompanied by increasing work pressure. These pressures and people’s poor health literacy might lead to a younger prevalence of ESRD (3). Data show that there are 120,000 new ESRD patients each year, 80% of whom are young and middle-aged in China (4). Moreover, the average age of MHD patients in China is more than 10 years younger than that in US and Japan (5). Uneven economic development, insufficient medical resources and uneven distribution, as well as low health literacy might be the reasons for the large number of young and middle-aged patients with ESRD in China. At the same time, due to the shortage of kidney resources and the high cost of treatment, most young and middle-aged patients still tend to be treated with MHD (6). MHD patients of young and middle-age have to change from playing the mainstay role of the family to the role of patients. They are more prone to maladjustment to the disease, which will affect the patients’ response with treatment and their health-related quality of life (HRQoL). Available studies have shown that socio-environmental, psycho-spiritual, and clinically relevant factors has resulted in a general reduction in HRQoL in young and middle-aged people with MHD (7, 8).

HRQoL refers to the self-assessment of health status in terms of physical, mental, social functioning based on personal experience and perceptions, reflecting the influence of disease and health on quality of life (QoL) (9). The level of HRQoL is important in guiding clinical decisions and has become a prognostic indicator and a survival indicator (10). A systematic review showed that suicidal behavior is closely related to HRQoL, that the lower the HRQoL, the higher the risk of suicidal behavior (11), and that this process is moderated by abnormalities in the hypothalamic–pituitary–adrenal (HPA) axis (12). MHD patients typically report poor HRQoL (13) and earlier studies have found that the total HRQoL scores of MHD patients are lower than abdominal dialysis patients (14), renal transplant patients (15), and patients with other chronic diseases (16). It has been well evidenced that hemodialysis impairs HRQoL in patients with CKD (17). How to help young and middle-aged patients reduce psychological distress, improve HRQoL and return to society to the greatest extent during MHD treatment has become a hot research issue in recent years. Thus, it is called for to have a deeper understanding of HRQoL in MHD patients of young and middle-age.

Many studies have shown that the socioeconomic status (SES) is significantly positively correlated with HRQoL, and SES is an important factor influencing individual QoL (18, 19). Nevertheless, few researches have specifically focused on the relationship between SES and HRQoL of MHD patients in young and middle age, and most of the existing studies only analyzed the direct link between the two, without focusing on the process and specific mechanisms of the impact of SES on HRQoL (20). Currently, most clinical studies on HRQoL in MHD patients have small sample sizes and are mostly single-center studies, with poor reproducibility of results. To sum up, this study aimed at exploring the relationship and mechanism between SES and HRQoL in young and middle-aged MHD patients through a multicenter cross-sectional study, and to provide a basis for the development of healthcare intervention programs to maintain or improve their HRQoL.

## 2. Background

SES is a social contextual culture that affects everyone who lives in it. SES is the position of an individual, family, or organization in the social structure, reflecting people’s ability to access or dispose of resources such as information, power, prestige, etc. (21). SES is usually measured using a combination of three objective indicators: level of education, economic income, and type of occupation (22). In medical sociology, SES is seen as a fundamental factor influencing health levels, and the health inequalities it causes are supported by a large body of research (23, 24). Studies have shown that SES differences lead to changes in the stress-sensitive HPA axis, which reduces the patient’s ability to control stress, and thereby increases negative emotions (e.g., anxiety, depression, and negative illness perceptions) (25) as well as suicidal behaviors (26). In addition, studies have found that patients with suicidal intent have higher levels of inflammatory factors compared to patients without suicidal intent (27), and higher levels of inflammation will also lead to a significant increase in drug resistance (28), which will impede the therapeutic efficacy of medications, thus seriously affecting the HRQoL of patients. The correlation between SES and health is also presented in the MHD population, with higher SES predicting good HRQoL scores (29). MHD patients in young and middle age shoulder the burden of family and social responsibilities, and regular dialysis treatment as well as reduced work capacity would lead to unemployment, causing their families’ SES to plummet and resulting in a lower QoL (30). Many researches have shown the association between SES and HRQoL in MHD patients, but relatively few studies have comprehensively explained the theoretical mechanisms by which SES affects HRQoL. Although certain factors of SES are difficult to change, the negative impact of a disadvantaged family background on HRQoL can be reduced through nursing interventions to regulate its psychosocial mechanisms and give full play to the patient’s initiative.

SES is not only an independent predictor of HRQoL in MHD patients (31) but also the primary factor affecting patients’ illness perceptions (32) and social functioning (33). The effect of SES on patients’ HRQoL is not always straightforward and is often mediated by psychological factors (34). The Self-Regulatory Model (SRM), states that illness perception, as a psychological representation, is central to an individual’s understanding, processing and ultimately coping with illness, reflecting the individual’s emotional responses and beliefs when facing health threats, thus guiding the patient’s coping style and influencing health outcomes (35). It was found that illness perception is a significant predictor of QoL for hemodialysis patients (36). Previous research has shown an intimate relationship between illness perception and coping styles, emotion regulation, social functioning, therefore had a significant effect on patients’ illness prognosis and QoL (37). It was previously found that the more intense the patient’s negative illness perceptions, the higher the incidence of social dysfunctions (38). Social functioning include both social roles and social interactions, and each person exhibits social behaviors according to his or her role in social life, and these behaviors and activities are the key elements of people’s social life (39). Researches have demonstrated that social functioning, as an important factor, can influence QoL in MHD patients (40). A normal return to work and normal interpersonal interaction can help patients regain confidence in their lives, reclaim a sense of social belonging, enhance their sense of self-worth, reduce psychological stress, enable them to focus more positively on themselves, take better care of themselves and improve their HRQoL (41). Nevertheless, under the combined pressure of economic stress and associated comorbidities (42), the positive psychological defenses of MHD patients can easily be breached, resulting in negative psychology such as panic, anxiety and depression, leading to deterioration in social role adaptation and social functioning, which seriously affects their HRQoL. In summary, based on previous studies, there are correlations between SES, illness perception, social functioning and HRQoL.

In Chinese cultural context, there are significant differences in the SES and social psychology of male and female. Studies at home and abroad have demonstrated that (43, 44) women’s health status is weaker than men’s due to their weaker SES, resulting in a disadvantageous access and utilization of health services for the female population. Significant gender differences was found in illness perception of people with chronic diseases (45). Also, previous studies have shown that male has a higher incidence of social functioning deficits than female. This suggests that the effect of SES on HRQoL in MHD patients may vary by gender.

**Figure 1:**
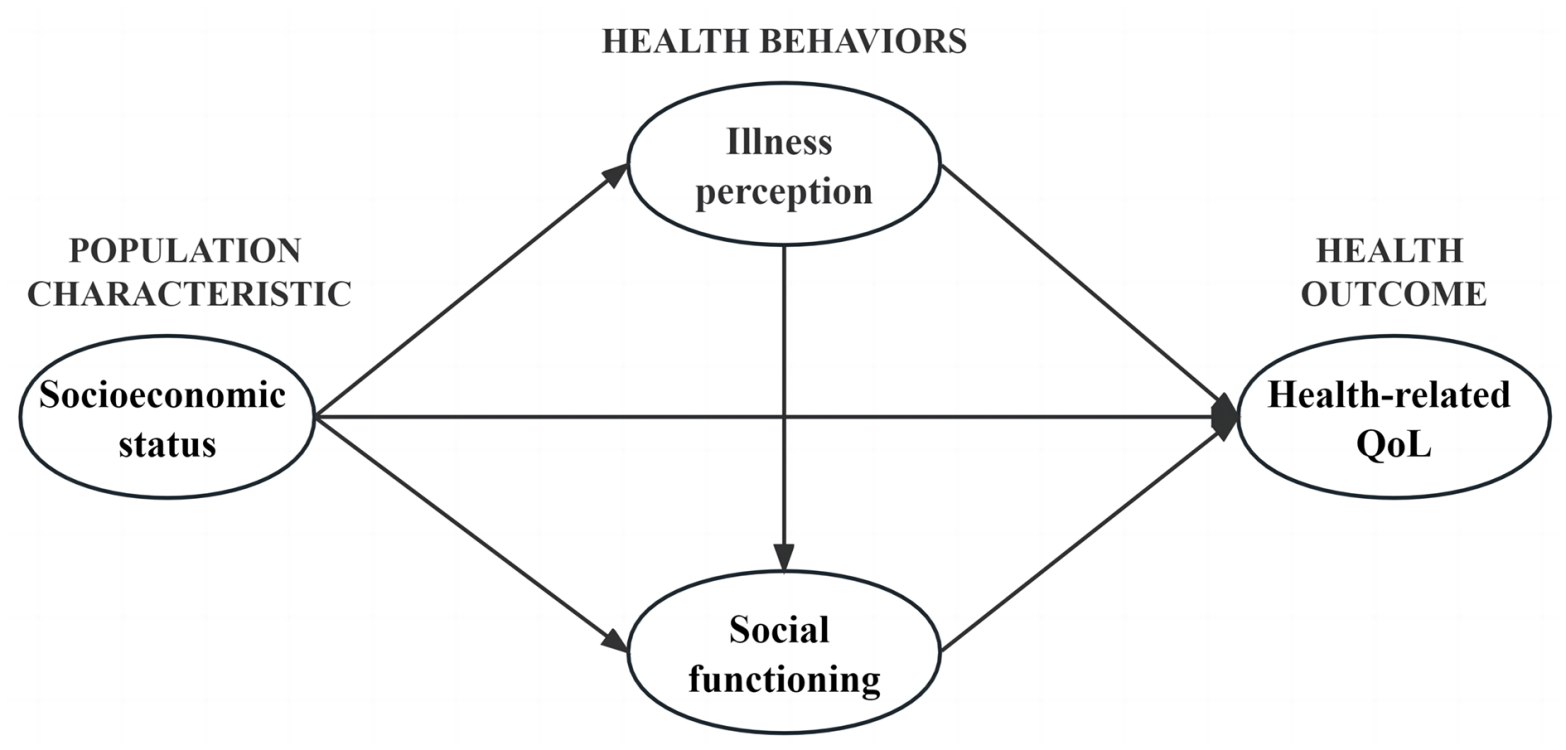
Theoretical hypothesis model.

The latest Andersen’s behavioral model provides a clearer explanation of the relationship between individual SES and health. From a cross-sectional perspective, environmental factors and population characteristics, as antecedents of health outcomes and health behaviors, could affect health outcomes both indirectly by influencing health behaviors and directly (46). Guided by this theory, this study considers health behaviors (illness perception and social functioning) as mediating variables to explore the relationship between population characteristic (SES) and health outcome (HRQoL) and the moderating effect of gender on the mediating model, with the aim of providing a theoretical basis for proposing medical care interventions to improve HRQoL in young and middle-aged patients. Therefore, our research hypotheses are as follows (see Figure 1):

*H1*: SES will significantly predict HRQoL.

*H2*: SES will influence HRQoL through the mediating effect of illness perception.

*H3*: SES will influence HRQoL through the mediating effect of social functioning.

*H4*: Illness perception and social functioning will jointly play an intermediary role in the association between SES and HRQoL.

*H5*: There may be a moderating effect of gender in the association between SES, illness perception, social functioning and HRQoL.

## 3. Methods

### 3.1. Study design

This study was a multicenter cross-sectional observational survey which adhere to the guidelines of the Strengthening the Reporting of Observational Studies in Epidemiology (STROBE; see Supplementary File S1).

### 3.2. Patients and setting

**Figure 2:**
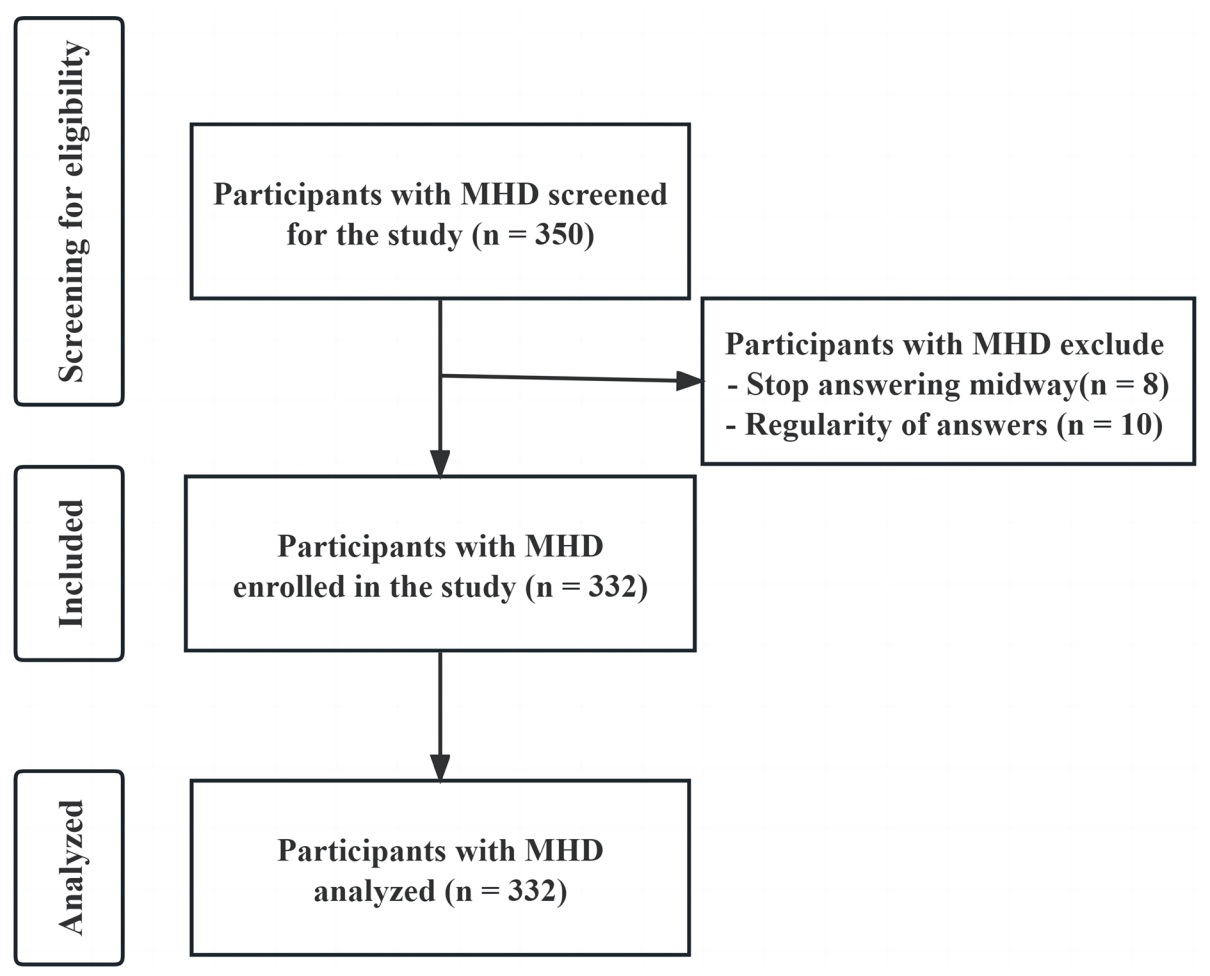
Flow chart of the study sample.

Young and middle-aged patients were selected through a convenience sampling method from hemodialysis centers of The First Affiliated Hospital of Guangzhou University of Chinese Medicine, Guangdong Second Hospital of Traditional Chinese Medicine, Guangzhou Hospital of Traditional Chinese Medicine, and Guangzhou Hospital of Integrated Traditional Chinese and Western Medicine, from June to December 2022. The following were inclusion criteria: (a) patient met the diagnostic criteria of stage 5 chronic kidney disease in the clinical guidelines of the US Kidney Disease Prognosis Quality Initiative (47); (b) Regularly receiving MHD treatment≥3 months; (c) Patients aged between 18 to 59 years old; (d) Ability to read and communicate in writing and orally; (e) Informed consent and voluntary participation in this survey. The following were exclusion criteria: (a) Patients with other serious diseases or malignant tumors; (b) Patients with cognitive dysfunction or mental illness; (c) Patients with visual, hearing, and speech impairments. Initially, 350 patients were selected for the study. 8 patients quit due to emotional distress or fatigue, and 10 participants responded with regularity. At the end, the sample included 332 MHD patients (participation rate = 94.9%; Figure 2).

### 3.3. Sample size

The sample size calculation was conducted by G*Power 3.1.9.7 software (48). With effect size (0.15), α error probability (0.05), power (0.95), and 21 predictors (three for SES, eight for illness perceptions, and ten for social functioning), we calculated the sample size to be at least 226 participants. The most appropriate sample size for using structural equation modeling (SEM) is 100–400 (49). The final number of participants in this study was 332 cases, which also met the criteria for using SEM.

### 3.4. Data collection and ethical considerations

Uniformly trained researchers collected the data through interviews, and used identical instructional language to guide patients to fill out the questionnaires during the survey. The purpose, significance, and confidentiality of the study were explained, so as to ensure authenticity of the study. Informed consent was obtained from all individual participants included in the study. All procedures of the study were ethically approved by the Ethics Committee of the authors’ hospital (K-2022-089).

### 3.5. Measurements

#### 3.5.1. Demographic and clinical variables

The variables included: age, gender, residence, marital status, medical insurance, dialysis status, comorbidities (cardiovascular disease, diabetes complications, mineral-Bone abnormalities, renal anemia), medication, and primary cause of illness.

#### 3.5.2. Socioeconomic status

In this study, the SES indicator was set according to the study by Pan et al. (50). The three variables, namely types of occupation, education level, and economic income of MHD patients at young and middle age were collected and assigned separately (See Supplementary File S2 for details of the assignments). Finally, the standard scores of these three variables were subjected to principal component analysis, which yielded one principal factor with an eigenroot greater than 1, explaining 56.5% of the variance, and the formula for calculating the indicator of comprehensive SES was obtained as follows: (0.793 × Z _occupation type_ + 0.717 × Z_monthly household income *per capita*_ + 0.744 × Z_education level_)/0.565, where 0.793, 0.717, and 0.744 represent the factor loadings of the three variables, respectively, and 0.565 represents the eigenroot of the first factor, with higher scores indicating higher SES. The SES of the patients in this study ranged from −5.27 to 9.82.

#### 3.5.3. The brief illness perception questionnaire

This study used the Chinese version of BIPQ to evaluate the cognitive and emotional representations of an illness in young and middle-aged MHD patients (51). The questionnaire consisted of 9 items, with a total score of 0 to 80. Higher scores represented the higher perceived threat of illness and more negative perceptions by patients. The scale is now commonly used in hemodialysis patients, with a measured Cronbach’s alpha coefficient of 0.67 (36). The Cronbach’s α coefficient for the scale in this study was 0.757.

#### 3.5.4. Social dysfunction screening scale

The scale was developed by the WHO (52) to assess the degree of social functioning of patients and is applicable to patients with all types of chronic diseases. It consists of 10 items, with a total score of ≥2 indicating deficits in social functioning. The scale demonstrated good validity and reliability in Chinese patients, with a retest reliability coefficient of 0.786 for young and middle-aged Chinese liver transplant recipients (53). The scale’s Cronbach’s α coefficient in this study was 0.781.

#### 3.5.5. The 12-item short form health survey

The scale is a simplified version of the MOS item short-form health survey (SF-36) and is primarily used to assess the HRQoL of patients (54). By assessing 8 domains, the 12-item scale measures the physical component summary score (PCS) and mental component summary score (MCS). The total HRQoL score is the average of the PCS and MCS scores, with a range from 0 to 100. Higher score indicates a better HRQoL for the patient. It has been proved that the Chinese version of SF-12 has good reliability (55, 56), and Cronbach’s α coefficient for the scale in this study was 0.624.

### 3.6. Data analysis

The outcomes were processed with SPSS version 25.0® and AMOS 28.0.

#### 3.6.1. Primary analysis

The reliability of the measuring tools was evaluated using Cronbach’s alpha. Participant demographic and clinical characteristics were tested with descriptive statistics. Furthermore, the samples were inspected for normality with skewness and kurtosis. To make comparison between the groups, a T-test or one-way ANOVA was applied. The presence of multicollinearity was determined using Variance inflation factor (VIF), tolerance, and Pearson’s correlation coefficient. The multivariate linear regression model was used to assess the impacts of social demographic characteristics and clinical variables, illness perception, as well as social functioning on HRQoL among MHD patients.

#### 3.6.2. Structural equation modeling analysis

The relationship between the latent factors in the hypothetical theoretical model was assessed through the SEM structural model. In the SEM, the maximum likelihood method was applied to determine the interrelationships and parameters among the variables. The bootstrap mediation effect test method was applied to inspect the mediating effects, set the sampling frequency to 5,000 times, and set the confidence intervals (CI) to 95%. A mediating effect exists if the 95% CI contains no 0. Then, the fit relationship between the hypothetical model and the data was tested by calculation of the fit indices. Finally, the moderating effect of gender was tested using a multi-group structural equation model. When modeling structural equations, because of the large amount of items in the existing scale, if the original items are directly used for modeling, the model structure may be more complex, the fitting degree is poor, and the parameter estimation deviation is high (57). From the modeling perspective, it has been proved that the item wrapping method could stabilize parameter estimates and improve model fit; from the measurement perspective, the advantage is to enhance the commonality of indicators and reduce random errors (58). Therefore, this study used item wrapping to enhance the fit and accuracy of the model. BIPQ was converted into three item packs (I1, I2, and I3), and ten items of SDSS were also converted into three item packs (S1, S2, and S3). The Harman single-factor test was applied to assess the common deviation before data analysis, thereby enhance the rigor of the study. The findings indicated that there were 9 factors with characteristic roots were more than 1, and the variation explained by the first factor was 24.13% (less than 40%), showing that there was no significant common.

## 4. Results

### 4.1. Participants’ demographic and disease-related characteristics

Three hundred thirty-two MHD patients (223 men and 99 women), with a mean age of 46.21 ± 9.49 years old, submitted complete questionnaires. Most of the patients were married (*n* = 251, 75.6%), living in cities and towns (*n* = 246, 74.1%), with medical insurance (*n* = 326, 98.2%), suffering from 3 to 4 kinds of chronic comorbidities (*n* = 168, 50.6%), and regularly taking 3 to 4 kinds of drugs (*n* = 179, 53.9%). 26.2% of participants had kidney disease due to hypertension. Regarding the duration of hemodialysis, 158 participants (47.6%) had hemodialysis duration ranging from 12 to 60 months. The frequency of dialysis was mainly 3 times a week, and the time of dialysis was mainly during daytime (Table 1).

**Table 1:** Demographic and clinical characteristics of participants (*N* = 332).

Variables	Categories	*N* (%)	HRQoL Mean ± SD	*t/F*
Gender	Male	233 (70.2)	57.09 ± 12.49	0.15
	Female	99 (29.8)	56.87 ± 12.00	
Age (years)	18–44	121 (36.4)	58.68 ± 11.98	1.86
	45–59	211 (63.6)	56.07 ± 12.45	
Marital status	Single	64 (19.3)	57.57 ± 12.65	0.70
	Married	251 (75.6)	57.15 ± 12.08	
	Divorced	11 (3.3)	51.99 ± 15.42	
	Widowed	6 (1.8)	55.21 ± 14.71	
Residence	Towns	246 (74.1)	58.19 ± 12.44	2.96*
	Rural	86 (25.9)	53.67 ± 11.41	
Medical coverage	Self-financed	3 (0.6)	45.31 ± 6.25	1.29
	Resident health insurance	134 (40.4)	56.31 ± 12.59	
	Employee medical insurance	192 (57.8)	57.73 ± 12.06	
	Commercial insurance	3 (0.9)	55.21 ± 20.09	
Duration of HD (months)	3 ~ 6	39 (11.7)	57.09 ± 13.19	0.49
	6 ~ 12	39 (11.7)	56.25 ± 11.44	
	12 ~ 60	158 (47.6)	56.44 ± 12.67	
	≥60	96 (28.9)	58.27 ± 11.83	
Dialysis frequency(times/week)	<3	42 (12.7)	60.34 ± 11.62	1.87
	≥3	290 (87.3)	56.54 ± 12.37	
dialysis time period	Daytime	283 (85.2)	56.45 ± 12.23	−2.05*
	Evening	49 (14.8)	60.33 ± 12.54	
Number of comorbidities	1 ~ 2	48 (14.5)	62.79 ± 11.27	10.89**
	3 ~ 4	168 (50.6)	57.79 ± 11.97	
	≥5	116 (34.9)	53.52 ± 12.27	
Number of medications	1 ~ 2	46 (13.9)	58.76 ± 11.33	2.54
	3 ~ 4	179 (53.9)	57.86 ± 12.46	
	≥5	107 (32.2)	54.86 ± 12.34	
Primary cause	Glomerulonephritis	38 (11.4)	60.32 ± 11.75	2.80*
	Diabetic nephropathy	56 (16.9)	51.53 ± 9.85	
	Hypertensive nephropathy	87 (26.2)	57.29 ± 13.65	
	Polycystic kidney	11 (3.3)	60.94 ± 10.80	
	Obstructive nephropathy	7 (2.1)	58.71 ± 11.30	
	Undetermined etiology	60 (18.1)	56.85 ± 12.36	
	Other	73 (22.0)	58.58 ± 12.03	

HD, hemodialysis; HRQoL, health-related quality of life; SD, standard deviation. *p < 0.05, **p < 0.01.

Regarding the SES, the education level of the participants in the sample is mainly middle school (*n* = 134, 40.4%). For financial status, 137 (41.3%) declared a household *per capita* monthly income of 4,001–6,000 Renminbi (RMB) per month, which belongs to the middle income group in China. According to the criteria for occupational classification, the study divided occupations into five grades, of which 225 (67.8%) participants were casual workers, unemployed, or unskilled and agricultural working class (Table 2).

**Table 2:** SES characteristics of participants (*N* = 332).

Variables	Categories	*N*(%)	HRQoL Mean ± SD	*t/F*
Occupation	Casual worker / Unemployed /Unskilled and agricultural working class	225 (67.8)	53.47 ± 11.50	20.96^**^
	Workers / Self-employed / Skilled worker	47 (14.2)	60.67 ± 10.64	
	Grassroots managers /Service industry workers / General professional and technical staff	49 (14.8)	66.80 ± 8.81	
	Middle management / Middle-level professional and technical staff	8 (2.4)	72.27 ± 7.17	
	Senior management staff	3 (0.9)	66.15 ± 23.66	
Monthly household income *per capita*(RMB)	<2,000	10 (3.0)	56.09 ± 12.14	8.71^**^
	2,000–4,000	126 (38.0)	53.79 ± 12.44	
	4,001–6,000	137 (41.3)	57.32 ± 11.56	
	>6,000	59 (17.8)	63.37 ± 11.57	
Education level	Primary school and below	79 (23.8)	53.78 ± 12.05	
	Middle school	134 (40.4)	56.72 ± 13.02	4.22^**^
	High school or secondary school	67 (20.2)	57.42 ± 10.78	
	Junior college	36 (10.8)	60.89 ± 11.50	
	≥College	16 (4.8)	65.23 ± 10.64	

SES, socioeconomic status; HRQoL, health-related quality of life; SD, standard deviation; RMB, Renminbi. *p < 0.05, **p < 0.01. For monthly household income *per capita*, <2,000 represents the low income families, 2,000–4,000 represents the lower-middle income families, 4,001–6,000 represents the middle income families, >6,000 represents the high income families in China.

### 4.2. Descriptive statistics and normality of research variables

Significant differences existed between male and female patients on the means of SES and social functioning, and non-significant differences on other variables. Considering that the absolute value of skewness for all studied variables ranged from 0.06–0.88, and the kurtosis ranged from 0.19–0.83, which indicated that the data conform to a multivariate normal distribution (Table 3).

**Table 3:** Descriptive statistics of study variables (*N* = 332).

Variables	Mean ± SD	Male (Mean ± SD)	Female (Mean ± SD)	*t*	Skewness	Kurtosis
Population characteristic						
SES	0.05 ± 3.03	0.29 ± 3.07	−0.50 ± 2.85	2.19*	0.88	0.62
Health behaviours						
Illness perception	51.71 ± 5.85	51.58 ± 5.72	52.00 ± 6.17	−0.59	−0.06	−0.19
Social functioning	3.64 ± 2.93	3.44 ± 2.96	4.13 ± 2.80	−1.98*	0.46	−0.83
Health outcome						
HRQoL	57.02 ± 2.93	57.09 ± 12.49	56.87 ± 11.99	0.15	0.12	−0.78

SES, socioeconomic status; HRQoL, health-related quality of life; SD, standard deviation. *p < 0.05.

### 4.3. Correlations of research variables

There were significant correlations between SES, illness perception, social functioning and HRQoL in young and middle-aged patients on MHD, which provided the prerequisites for the subsequent multiple mediated effects analysis (Table 4). The tolerance was between 0.57 to 0.71, the VIF ranged from 1.40 to 1.76, and the correlation coefficient between variables was between −0.64 and 0.51, showing no significant multicollinearity between the measured variables.

**Table 4:** Correlation analysis between SES, illness perception, social functioning and HRQoL (*N* = 332).

Variables	1	2	3	4	Tolerance	VIF
1. SES	1				0.62	1.64
2. Illness perception	−0.36**	1			0.71	1.40
3. Social functioning	−0.55**	0.51**	1		0.57	1.76
4. HRQoL	0.44**	−0.59**	−0.64**	1	-	-

SES, socioeconomic status; HRQoL, health-related quality of life; VIF, variance inflation factor. **p < 0.01.

### 4.4. Multivariate linear regression analysis of factors affecting the HRQoL of young and middle-aged MHD patients

The variables associated with HRQoL in the univariate analysis were included in the model. Multivariate stepwise linear regression analysis was performed with residence, dialysis time period, chronic comorbidities and number of long-term medications as control variables, while SES, illness perception, and social dysfunction as independent variables, and total SF-12 score as a dependent variable, aiming to determine the predictive effect of HRQoL in young and middle-aged MHD patients (Table 5). The outcomes of the Model I indicated that residence (*β* = −3.78, *p* < 0.05) and comorbidities (*β* = −3.73, *p* < 0.01) demonstrated a notable negative predictive effect on HRQoL. In Model II, SES (*β* = 1.62, *p* < 0.01) significantly and positively predicted HRQoL, so *H1* holds. Based on Model II, a significant negative effect on patients’ HRQoL was found in Model III with illness perception (*β* = −1.02, *p* < 0.01) as the independent variable. In Model IV, when participation in SES, illness perception and social dysfunction were entered into the regression equation simultaneously, the predictive effect of illness perception on HRQoL remained significant, and social dysfunction (*β* = −1.77, *p* < 0.01) also significantly and negatively predicted HRQoL. However, the positive predictive effect of SES on HRQoL was decreased and non-significant (*β* = 0.36, *p*>0.05), suggesting that illness perception and social functioning play a fully mediating role between SES and HRQoL. Furthermore, as shown in Table 5, Model IV had the largest adjusted *R*^2^ compared to the first three models, suggesting that SES, illness perception and social functioning together have greater explanatory power for HRQoL.

**Table 5:** Multivariate linear regression analysis of factors affecting the HRQoL (*N* = 332).

Model	Variables	*β*	SD	Standardized *β*	*F* value	Adjust *R*^2^
Model I	Residence	−3.78*	1.50	−0.13	8.16**	0.08
	Dialysis time period	2.77	1.85	0.08		
	Number of comorbidities	−3.73**	1.01	−0.20		
	Number of medications	−1.55	1.02	−0.08		
Model II	Residence	−0.37	1.47	−0.01	17.88**	0.20
	Dialysis time period	1.10	1.74	0.03		
	Number of comorbidities	−1.80	0.98	−0.10		
	Number of medications	−1.78	0.95	−0.10		
	SES	1.62**	0.23	0.40		
Model III	Residence	0.28	1.28	0.01	38.35**	0.40
	Dialysis time period	1.78	1.51	0.05		
	Number of comorbidities	−0.50	0.85	−0.03		
	Number of medications	−1.23	0.82	−0.07		
	SES	1.02**	0.20	0.25		
	Illness perception	−1.02**	0.10	−0.49		
Model IV	Residence	0.16	1.16	0.01	49.07**	0.50
	Dialysis time period	0.29	1.39	0.01		
	Number of comorbidities	0.11	0.78	0.01		
	Number of medications	−1.49*	0.75	−0.08		
	SES	0.36	0.20	0.09		
	Illness perception	−0.71**	0.10	−0.34		
	Social functioning	−1.77**	0.21	−0.42		

HRQoL, health-related quality of life; SD, standard deviation; SES, socioeconomic status. *p < 0.05, **p < 0.01.

### 4.5. Mediation model construction

**Figure 3:**
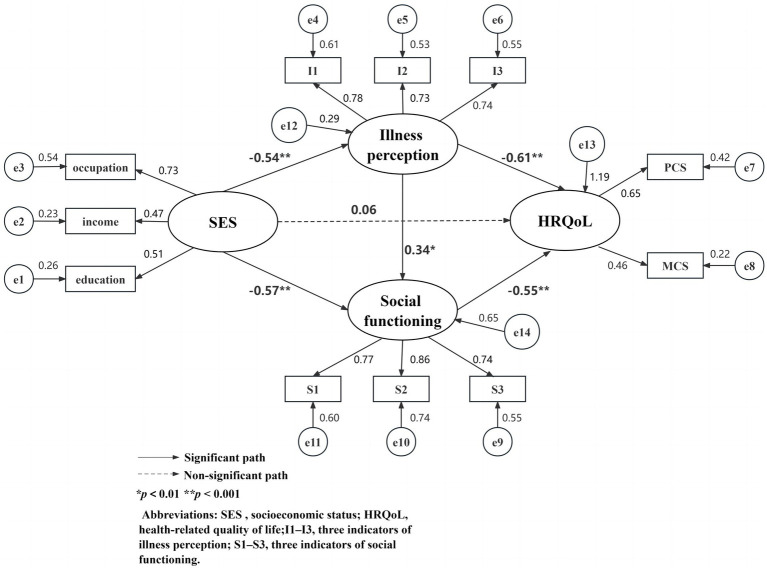
The final SEM model.

Based on Andersen’s behavioral model and the multilevel regression analysis outcomes previously demonstrated, the mediation model was constructed using AMOS 28.0 analysis software with SES as the antecedent variable, illness perception and social functioning as mediating variables, and HRQoL as the outcome variable. The correlations and effect paths of variables are presented in the final output model (Figure 3), justifying research hypotheses *H2*, *H3*, and *H4*. The fitted indicators of the model were *χ*^2^/df = 1.897<3, SRMR = 0.036 < 0.04, RMSEA = 0.052<0.08, CFI = 0.974>0.9, NFI = 0.948>0.9, TLI = 0.963>0.9, which met the criteria of excellent model fit.

### 4.6. Mediation model validation and effect analysis

As shown in Figure 3, the direct predictive effect of SES on HRQoL was not significant (*p* > 0.05) after bringing in the two intermediary variables: illness perception and social functioning; illness perception (*β* = −0.61, *p* < 0.001) and social dysfunction (*β* = −0.55, *p* < 0.001) each had a notable negative predictive effect on HRQoL, both consistent with the outcomes of multiple linear regression analysis. Besides, SES was an important negative predictor of illness perception (*β* = −0.54, *p* < 0.001) and social dysfunction (*β* = −0.57, *p* < 0.001). Social dysfunction was positively predicted by illness perception (*β* = 0.34, *p* = 0.003).

Table 6 shows the specifics of the direct and indirect associations between SES and HRQoL. SES had total and indirect effects as 0.79 [95% CI (0.028, 0.985)] and 0.74 [95% CI (0.628, 1.391)], respectively, on HRQoL. The two specific mediating effects of illness perception and social dysfunction were both statistically significant, with the indirect effects of SES on HRQoL via illness perception and social dysfunction being 0.33 [95% CI (0.204, 0.494)] and 0.31 [95% CI (0.148, 0.570)], making up 41.06% and 38.91% of the total effect, respectively. The chain indirect effect of illness perception and social dysfunction was 0.10 [95% CI (0.040, 0.178)], making up 12.59% of the sum. The direct effect between SES and HRQoL was not statistically significant (*p* > 0.05).

**Table 6:** Direct and indirect effects of SES and HRQoL.

	Path	Effect	SD	LLCI	ULCI	Relative mediation effect (%)
Direct effects	SES-HRQoL	0.06	0.17	−0.320	0.363	7.43
Indirect effects	SES-IP-HRQoL	0.33^**^	0.08	0.204	0.494	41.06
	SES-SD-HRQoL	0.31^**^	0.11	0.148	0.570	38.91
	SES-IP-SD-HRQoL	0.10^*^	0.04	0.040	0.178	12.59
Total indirect effects		0.74^**^	0.15	0.628	1.391	92.57
Total effects		0.79^**^	0.09	0.028	0.985	—

HRQoL, health-related quality of life; SES, socioeconomic status; IP, illness perception; SD, social dysfunction; SD, standard deviation; LLCI, lower limit confidence intervals; ULCI, upper limit confidence intervals. *p < 0.01, **p < 0.001.

### 4.7. Analysis of gender differences in mediation model

Gender was used as a moderating variable for the multi-group comparisons to test whether the multiple mediation model would be affected. First, the model was tested separately for the male and female patients samples and the results showed (see Table 7) that the model fit well for both male and female patients and could be compared across groups. Next, the unconstrained model (M0) and the model with equal structural weights (M1) were set and the results showed (see Table 7) that the two models M0 and M1 fit well with no significant difference between them (△*χ*^2^ = 11.506, △df = 6, *p* = 0.747>0.05), suggesting that gender cannot play a moderating role in the multiple mediation model.

**Table 7:** Multi-group structural equation model fit indices.

	*χ* ^2^	df	*χ*^2^/df	GFI	AGFI	RMSEA	CFI	IFI	NFI
M male	62.772	38	1.652	0.953	0.918	0.053	0.975	0.900	0.941
M female	69.978	38	1.842	0.891	0.811	0.093	0.906	0.910	0.823
M 0	132.928	76	1.746	0.934	0.885	0.484	0.958	0.959	0.909
M 1	144.434	82	1.761	0.929	0.885	0.484	0.954	0.955	0.901

M, model; M0, the unconstrained model; M1, the model with equal structural weights.

## 5. Discussion

Based on Andersen’s behavioral model, we examined a serial multiple mediation model with illness perception and social functioning in the association between SES and HRQoL. Specific findings and analyzes of the study are as follows.

### 5.1. Current status of SES, illness perceptions, social functioning and HRQoL in young and middle-aged people with MHD

Compared with the domestic norm, the HRQoL scores of young and middle-aged MHD patients were lower in this study, which was consistent with previous study (59), suggesting that the HRQoL of young and middle-aged MHD patients was at a low level. The goal of MHD treatment is not only to improve the clinical symptoms of patients and prolong their survival time, but also to improve their QoL, which suggests that improving the HRQoL of patients is a long-term exploration for medical professionals. In this study, young and middle-aged MHD patients’ SES was generally low-characterized by low educational attainment, lack of employment and heavy financial burden, which is consistent with the findings of Modi et al. (60). Due to frequent hemodialysis treatment and its associated symptoms (8), most hemodialysis patients quit their work or cut down their working hours after starting dialysis treatment, resulting in unfulfilled career goals, which greatly affects their QoL and personal development, and also places a heavy financial burden on their families and society, greatly reducing their SES. Therefore, medical professionals should focus on the low SES group of young and middle-aged MHD. The level of illness perceptions in young and middle-aged MHD patients was in the upper middle range, which is comparable to some results reported in the literature (61), but much higher than scores for other chronic diseases (62). Appropriate illness perceptions can promote a positive outlook on illness and its treatment, but excessive illness perceptions can lead to incorrect or negative perceptions of illness and treatment, and affect long-term QoL (63). This suggests that in the treatment of MHD, medical professionals should promptly assess patients’ illness perceptions, grasp their concerns and understanding of the illness, and enhance health education to reduce their negative perceptions. In addition, the current state of social functioning of young and middle-aged MHD patients was not promising, and 68.6% of patients had varying degrees of social dysfunctions. The specific manifestations are decreased work and professional ability, social withdrawal, less social activities inside and outside the family, lack of interest and concern for the outside world, and lack of responsibility and planning. This may be due to the fact that dietary restrictions, various complications, and dependence on hemodialysis for survival have caused patients to position themselves in a patient role, both psychologically and physically, thus reducing their social participation and level of social engagement. A study has shown that good social functioning is vital to the physical and mental health of patients and the treatment of their illnesses (64). Therefore, improving social functioning of young and middle-aged MHD patients and enabling them to adapt to the life changes brought about by hemodialysis as soon as possible is important for improving long-term treatment and HRQoL.

### 5.2. The influence mechanism of SES on HRQoL

This study focused on the effect of SES on HRQoL and analyzed the internal mediating mechanism of illness perception and social functioning with residence, dialysis duration, and number of chronic comorbidities as well as long-term-medications as control variables. The findings suggested that SES has a significant positive influence on HRQoL of young and middle-aged MHD patients, which is consistent with previous findings that employment status, *per capita* household income, and literacy will affect HRQoL (19, 65). The Andersen’s behavioral model states that SES is an important factor affecting health outcomes for vulnerable groups (46). Multiple studies have shown that low SES in patients with MHD is consistently associated with impaired HRQoL (20, 66). SES is closely related to the degree of HRQoL in young and middle-aged MHD patients, but there are few studies on the internal mediating mechanism of SES on HRQoL. In this study, it was noteworthy that the direct predictive effect of SES on HRQoL was insignificant after introducing the two intermediary variables of illness perception and social functioning. These two intermediary variables could take effect separately or jointly, thus established the chain effect of SES → illness perception → social functioning → HRQoL, which suggests that illness perception and social functioning play a fully mediating role between SES and HRQoL, and this result also corroborates the existence of mediating modes in the effect of SES on an individual’s health (46). This result may suggest that improving the objective material basis alone does not improve HRQoL in young and middle-aged MHD patients and that other individual factors influenced by SES (illness perception, social functioning) are more closely related to HRQoL.

### 5.3. Mediating effect of illness perception

The research results showed SES can affect the HRQoL of young and middle-aged MHD patients through a separate mediating effect of illness perception. This result supports the view of the Andersen’s behavioral model that the propensity trait of SES needs to interact with positive resources within the individual to have an impact on HRQoL in young and middle-aged MHD patients. Illness perception reflects the psychological representation of MHD patients toward the disease, and affects the patients’ cognition and coping behavior toward the disease. The findings that illness perception has a direct effect on HRQoL and that other factors are indirectly related to HRQoL through the mediation of illness perception is consistent with the results of another study (67) and confirms *H2* of this study. Reserve capacity Model also states that groups with low SES experience more stress from internal and external sources, which depletes their own psychosocial resources, therefore leads to more negative emotions and impairs QoL (68). The more negative illness perceptions among hemodialysis patients of lower SES, the lower the overall HRQoL score, a finding consistent with data from the study reported by Chen et al. (36). Patients of low SES tend to perceive more severe disease outcomes, believe their disease will last longer, have more symptoms and more emotional reactions because of their disease, and conversely, those of higher SES have stronger beliefs about disease status control and better disease understanding (34). This may be due to differences in SES affect patients’ ways of thinking, interpersonal skills and resilience to stress, as well as their perception and acceptance of the facts of the illness, such that individuals may have positive illness perceptions (seeking relevant information, coping positively, increasing confidence, etc.) or negative illness perceptions (avoidance, denial, negative emotions, etc.) for the same health problem, which could bring positive or negative influence to their health outcomes, respectively.

### 5.4. Mediating effect of social functioning

Social functioning is also a mediating variable between SES and HRQoL, which confirms *H3*. Social Cognitive Theory states that social class shapes the environment in which individuals live, causing them to develop a social cognitive style appropriate to their class, and that this stable social cognitive style in turn influences their psychological and behavioral responses (69). As a result, there are significant differences in the social cognitive styles and behaviors of individuals of different SES (70). However, the relationship between sociodemographic factors and social functioning remains inconsistent and still needs further verification. In this study, the higher the SES of the patients, the lower the likelihood of social dysfunctions and the higher their level of HRQoL, which supports the Andersen’s behavioral model that population characteristics (SES) can influence health outcomes (HRQoL) through the mediation of health behaviors (social functioning).

Sound social functioning is an objective reflection of an individual’s QoL; and defective social functioning can lead to disorder in the individual’s social function and social behavior dysfunction (71). Enabling people with MHD to function and behave socially in accordance with their role in society can help build confidence in overcoming their illness and is essential to improving their QoL (33). Thus, social functioning becomes one of the intermediate mechanisms linking SES and HRQoL, i.e., differences in SES can lead to inequalities in HRQoL through social functioning.

### 5.5. The chain intermediary of illness perception and social functioning

This study showed that SES has an impact on HRQoL of young and middle-aged MHD patients through the chain intermediary effect of illness perception and social functioning, so *H4* of this study was confirmed. The result suggested that SES directly affects patients’ cognitive and emotional responses to illness, and that individuals with lower levels of SES have lower reasoning and cognitive control and are more likely to develop negative illness perceptions, leading to socially deficient behaviors and consequently impaired HRQoL, which mirrors the theoretical view of the Andersen’s behavioral model. Heavy family and social roles taken on by middle-aged youth. Once young and middle-aged people suffer from chronic diseases, in the face of multiple blows of disease torture and psychological trauma as well as economic pressure, the low SES patient group is more likely to cause emotional and cognitive behavioral abnormalities toward the disease, thus inducing negative emotions in patients and affecting their social participation (72). Meanwhile, altered physiological functions and partial physical deficits can lead to abnormal social roles and negative attitudes that inhibit behavior and expression in social interactions, thus preventing a successful return to the family and society and resulting in social dysfunction (64). Social dysfunction will lead to the formation of patients’ negative values and the aggravation of social behavior withdrawal, which will also affect the patient’s disease outcome, cause huge wealth loss to the family and society, increase the economic burden on the family and society, and make health-related diseases worse. Quality of life is severely impaired (73). Current research has identified that people with higher SES tend to cope with stress and regulate their emotions better, and that these strengths further contribute to their behavior and mindset in everyday life and to their physical health (34). Therefore, simultaneous interventions on illness perception and social functioning in young and middle-aged MHD patients are more conducive to HRQoL improvement.

### 5.6. The moderating role of gender

Although this study found significant gender differences in SES and social functioning in patients, there were no significant gender differences in the mediating role of illness perception and social functioning between patients’ SES and HRQoL, suggesting that gender does not have a significant moderating role in this multiple mediation model and that the mediating mechanism between SES and HRQoL is intrinsically similar between patients of different genders.

In conclusion, although there are characteristics of SES that cannot be easily changed, medical staff can pay close attention to the psychological resources of young and middle-aged MHD patients. According to positive psychology, to guide patients to correctly understand the disease, eliminate negative emotions, enhance the sense of belief in disease treatment, and promote patients to actively adapt to social roles and re-engage in social life, thus improving their HRQoL.

### 5.7. Implications for practice

The results of the present study suggest that that SES positively predicts HRQoL through the multiple mediating effects of illness perception and social functioning, i.e., higher SES predicts positive illness perception and good social functioning, thus contributing to the maintenance of good HRQoL, which provides a scientific and theoretical basis for developing medical care interventions to maintain or improve patients’ HRQoL from multiple perspectives. Specifically, nurses should encourage young and middle-aged MHD patients to return to work, and can promote the recovery of patients’ working ability by carrying out health education on MHD and work, formulating targeted vocational rehabilitation programs and conducting relevant vocational skills training, so as to improve their SES. The findings suggest that illness perception and social functioning play multiple mediating roles in SES affecting HRQoL, nurses should appropriately assess and monitor patients’ illness perception and social functioning and give timely and targeted interventions. Nursing interventions are of vital importance in shaping a patient’s illness perception (74). Pre-dialysis care should be enhanced by combining motivational interviewing with psycho-behavioral interventions, health education care and collaborative care models to reduce patients’ negative illness perceptions. During long term dialysis treatments, positive illness perceptions of patient can be built up through group management health education, patient exchange meetings, hope therapy, etc. and reinforced by practice in daily life. Therefore, nurses should enhance pre-dialysis care for patients by combining motivational interviewing with psycho-behavioral interventions, knowledge, belief and practice health education care and collaborative care models to reduce patients’ negative illness perceptions. During long term dialysis treatments, positive illness perceptions of patient can be built up through group management health education, patient exchange meetings, hope therapy, etc. and reinforced by practice in daily life. At the same time, nurses should develop and implement a comprehensive and dynamic care strategy for young and middle-aged MHD patients with social dysfunction to help them adapt well to the role change and promote the recovery of social function. Young and middle-aged MHD patients should not only actively participate in social activities, establish normal interpersonal and harmonious family relationships, and engage in work that is within their capacity, but also actively communicate with healthcare professionals to improve their overall understanding of the disease and take the initiative to reshape a positive and healthy way of thinking so as to improve their HRQoL.

### 5.8. Limitations

There are certain theoretical and practical implications of this study, but there are also some limitations. First, we cannot directly derive causal relationships from the cross-sectional design. Therefore, a longitudinal study design would be ideal to examine the complex dynamic effects of SES, illness perception, and social functioning on HRQoL. Second, this study used convenience sampling and self-reporting methods to collect data, which may lead to selection bias and reporting bias, therefore, the results of this study need to be applied with caution. In the future, data collection should combine with self-evaluation and others’ evaluation, and sampling methods can also be changed to validate the findings of this study. Thirdly, as there are many factors affecting the explanatory variables, and the SES analyzed in this study is only one aspect affecting patients’ HRQoL, the multiple mediator model developed is not the only mediator model, therefore there are many other explanatory and mediator variables that deserve to be tested in the future. Fourthly, SES was measured only based on the types of occupation, education level, and economic income in this study. More indices of SES should be used in future studies.

## 6. Conclusion

In summary, based on Andersen’s behavioral model, we constructed a model of the intrinsic mechanism of action between SES and HRQoL in young and middle-aged MHD patients, which provided a reference for explaining and intervening the effect of SES on HRQoL. Considering the important role of illness perception and social functioning in the SES-HRQoL linkage, clinical nurses should intervene in health differences due to SES in terms of illness perception and social functioning to assist in enhancing HRQoL levels in young and middle-aged MHD patients.

## Data availability statement

The raw data supporting the conclusions of this article will be made available by the authors, without undue reservation.

## Author contributions

QM made substantial contributions to conception and design, acquisition of data, analysis and interpretation of data, and wrote the manuscript. SX and JH contributed to data collection and drafting of the manuscript. XS has revised this manuscript critically for important intellectual content. GC and ZM contributed to data analysis and revising it critically for important intellectual content. YS and CW supervised the whole process and provided modification advice. All authors contributed to the article and approved the submitted version.
