# Supplementary material for: The association between socioeconomic status and health-related quality of life among young and middle-aged maintenance hemodialysis patients: multiple mediation modeling
Source: Front Psychiatry. 2023 Sep 19;14:1234553. doi: 10.3389/fpsyt.2023.1234553 (PMC10546310; doi:10.3389/fpsyt.2023.1234553)
Supplement: Supplementary file 2 [file Table_2.docx]

Supplementary File 2

Details of the assignment of socio-economic status indicators

| **Index** | **Level** | **Assignment** |
| --- | --- | --- |
| Monthly household income per  capita(RMB) | ＜2000 | 1 |
|  | 2000-4000 | 2 |
|  | 4001-6000 | 3 |
|  | ＞6000 | 4 |
| Education level | Primary school and below | 1 |
|  | Middle school | 2 |
|  | High school or secondary school | 3 |
|  | Junior college | 4 |
|  | ≥College | 5 |
| Occupation | Temporary workers, unemployed, non-working people, unskilled and agricultural working classes such as farmers. | 1 |
|  | Manual and self-employed workers, skilled and equivalent workers, such as construction workers and related personnel. | 2 |
|  | General management and general professional and technical staff, clerical staff, including those in the business services sector, clerical staff, e.g. salesmen, drivers, etc. | 3 |
|  | Middle management and middle-level professional and technical personnel, assistant professionals, including those specializing in various professional and scientific work in institutions of various economic components (including state organs, party organizations, national enterprises and institutions, collective enterprises and institutions and various non-public economic enterprises), such as teachers, doctors, technicians, etc. | 4 |
|  | Professional senior managers and senior professional technicians, professional supervisors, including leading cadres exercising actual administrative authority in party, government, public institutions and social organizations, senior and middle managers who are not owners in large and medium-sized enterprises, and the class of private enterprise owners, such as civil servants, company managers, foremen, etc. | 5 |
